# The structural changes of upper airway and newly developed sleep breathing disorders after surgical treatment in class III malocclusion subjects

**DOI:** 10.1097/MD.0000000000006873

**Published:** 2017-06-02

**Authors:** Ui Lyong Lee, Hoon Oh, Sang Ki Min, Ji Ho Shin, Yong Seok Kang, Won Wook Lee, Young Eun Han, Young Jun Choi, Hyun Jik Kim

**Affiliations:** a Department of Otolaryngology and Head & Neck Surgery; b Department of Oral and Maxillofacial Surgery; c Chung-Ang University College of Medicine, Department of Otorhinolaryngology, Seoul University College of Medicine, Seoul, South Korea.

**Keywords:** bimaxillary surgery, class III malocclusion, obstructive sleep apnea, posterior airway space, snoring

## Abstract

Bimaxillary surgery is the traditional treatment of choice for correcting class III malocclusion which is reported to cause an alteration of oropharyngeal structures and upper airway narrowing that might be a predisposing factor for obstructive sleep apnea (OSA). This study aimed to analyze sleep parameters in class III malocclusion subjects and ascertain the prevalence of snoring or OSA following bimaxillary surgery.

A total of 22 patients with Le Fort I osteotomy and mandibular setback for class III malocclusion were prospectively enrolled. All patients received endoscopic examination, cephalometry, 3-dimensional computed tomography (3D-CT), and sleep study twice at 1 month before and 3 months after surgery.

The patient population consisted of 5 males and 17 females with a mean body mass index of 22.5 kg/m^2^ and mean age of 22.1 years. No patients complained of sleep-related symptoms, and the results of sleep study showed normal values before surgery. Three patients (13%) were newly diagnosed with mild or moderate OSA and 6 patients (27%) showed increased loudness of snoring (over 40 dB) after bimaxillary surgery. According to cephalometric analysis and 3D-CT results, the retropalatal and retroglossal areas were significantly narrowed in class III malocclusion patients, showing snoring and sleep apnea after surgery. In addition, the total volume of the upper airway was considerably reduced following surgery in the same patients.

Postoperative narrowing of the upper airway and a reduction of total upper airway volume can be induced, and causes snoring and OSA in class III malocclusion subjects following bimaxillary surgery.

## 1 Introduction

Approximately 4% of the population has dentofacial deformities that induce cosmetic problems to the patients, and these deformities occasionally require surgical treatment to correct. The most common deformities that need a surgical correction are severe class II and class III malocclusion and vertical skeletal discrepancies in nongrowing patients.^[1,2]^ Class III malocclusions present with skeletal discrepancies, such as a prognathic mandible with or without a protruding maxilla, and class III malocclusion patients make up a large proportion of those seeking surgical-orthodontic treatment due to aesthetic and functional problems.^[3–9]^

Mandibular setback osteotomy has routinely been used as an orthognathic surgical procedure for mandibular prognathism and bimaxillary orthognathic surgery including mandibular setback, and maxillary backward movement procedures are commonly used to correct class III malocclusion.^[10]^ For patients with skeletal class III malocclusion, bimaxillary surgery can reposition the orofacial skeletal together with soft tissue components, such as the soft palate and the tongue base.

Bimaxillary surgery is an elective surgical procedure carried out mostly in young and healthy patients in the context of orthognathic therapy. Possible complications of bimaxillary surgery may be divided into early and late problems. Early complications consist of bad splits, excessive intraoperative bleeding, delayed wound healing, and neurosensory alteration after osteotomy.^[5,6]^ Some authors have noted that narrowing of the posterior airway space (PAS) is expected after bimaxillary surgery.^[7,8]^ As a consequence, patients who receive bimaxillary surgery might suffer from sleep-disordered breathing in particular, which is due to the narrowing of the PAS and airway collapse during sleep.^[9–11]^ Sleep breathing disorders (SBDs) including obstructive sleep apnea (OSA) can be counted as a late complication of bimaxillary surgery. OSA is a common sleep disorder characterized by airway collapse at multiple levels of the upper airway, causing reduction or cessation of airflow during sleep.^[12]^ It has been reported that both upper airway narrowing and increased airway resistance might be the main pathogenesis of OSA, leading to loud snoring, apnea, and systemic complications without proper treatment.^[13–19]^ The potential effect of bimaxillary surgery-induced PAS narrowing in the development of sleep-disordered breathing or OSA remains under debate, along with other controversial questions such as whether bimaxillary surgery causes changes in sleep parameters. There have been few functional assessments about the prevalence of OSA using pre- and postoperative sleep studies in patients with dentofacial deformities. In particular, the clinical correlation between PAS narrowing following surgery and development of snoring or sleep apnea in class III malocclusion subjects has not been definitively investigated.

Accordingly, we designed a prospective clinical study including sleep parameters and 3-dimensional (3D)-computed tomography (CT) in class III malocclusion subjects before and after bimaxillary surgery to assess the alterations of the PAS and total upper airway volume. The purpose of present study was to ascertain clinical correlations with the prevalence of snoring and OSA as the degree of skeletal movement following bimaxillary surgery.

## 2 Materials and methods

### 2.1 Study populations and surgical technique for collection of maloccusion

A total of 22 adult subjects who underwent bimaxillary surgery at Chung-Ang University Hospital (Seoul, Korea) from March 2014 to February 2015 voluntarily participated in the study. Sleep study and volumetric measurement were performed prospectively. Written informed consent was obtained from each participant, and the study complied with the Declaration of Helsinki. The institutional review board of Chung-Ang University Hospital approved this study (C2014148 [1344]).

We designed a prospective study to recruit subjects diagnosed with skeletal class III malocclusions and scheduled to have bimaxillary surgeries at the department of oral and maxillofacial surgery. Subjects who underwent bimaxillary surgery, had a body mass index above 30 kg/m^2^, or were aware of their OSA or snoring before surgery were excluded. In addition, subjects with severe septal deviation, chronic hypertrophic rhinitis, tonsil hypertrophy, respiratory diseases, asthma, and cigarette smoking history that could affect the development of snoring or OSA were not included in the present study.

For the primary treatment, all enrolled subjects received a Le Fort I osteotomies through a horizontal incision in the buccal sulcus from the first premolar to the premolar that resulted in a horizontal osteotomy line of at least 5 mm away from the root apices. The osteotomy was completed from the pyriform rim to the posterior extent of the zygomatic buttress in the lateral wall of the maxilla in a plane horizontal to Frankfort horizontal (F-H) plane. After fracture, the maxilla was passively positioned into the desired location utilizing an interocclusal interim splint. The plates were bilaterally placed at the pyriform rims and the zygomatic buttresses. Next, bilateral sagittal split ramus osteotomies were performed, and then medial ascending ramus and buccal vertical cuts were completed. The buccal vertical cut continued through the inferior border and included 3—4 mm of the lingual cortex. After chiseling, the medial and lateral cortical plates were separated. Each adjustable plate contained 3 holes, and screws were used for the rigid fixation of split segments.

### 2.2 Study design and sleep study

All subjects were required to complete a pre- and postoperative research survey for evaluation of snoring and OSA following surgery. Watch-PAT 200 (Itamar Medical Ltd., Caesarea, Israel) was used for the diagnosis of OSA, assessment of OSA severity, and measurement of snoring. The watch-PAT was also performed 1 month before and 3 months after surgeries, and the apnea-hypopnea index (AHI), lowest oxygen saturation, valid sleep time, and decibel (dB) of snoring were evaluated. We estimated the prevalence and severity of OSA through pAHI and defined the threshold of snoring as over 40 dB. To analyze sleep architectures, PAT studies were uploaded for automated analysis on a personal computer using the COMPACTFLASH reader provided with the PAT software (zzz_PAT version 1.5.44.7; Itamar Medical Ltd.).

### 2.3 Cephalometric analysis

Lateral cephalometric radiographs were performed and assessed PAS and mandibular plane to hyoid (MPH) before and after bimaxillary surgery in class III malocclusion subjects to assess the retropalatal and retroglossal airway narrowing.^[16]^

### 2.4 Cone beam computed tomogram for airway measurement

All patients underwent Cone beam computed tomograms (CBCT; 3D eXam, Kavo Dental GmbH, Biberach, Germany) before surgery (T0) and 3 months after surgery (T1). CBCT was taken with each patient in an upright position with the F-H plane parallel to the floor and with a centric occlusion.^[17]^ The CBCT data of the maxillofacial regions were obtained with a 0.4 mm voxel size and 512 × 512 matrices, using 120 kVp, 11 mA, 17.8 s scan time, and a 12-inch detector field. Patient data were stored in DICOM (Digital Imaging and Communications in Medicine) format and reconstructed into 3D images using software (Invivo5; Anatomage, San Jose, CA).

**Figure 1:**
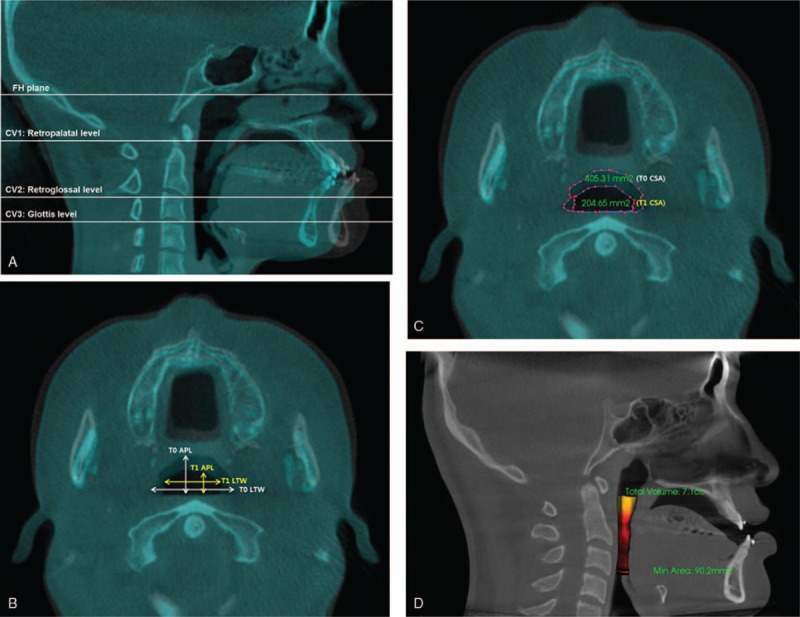
Measurement of airway diameters or upper airway volume using cone beam CT (CBCT). The pre- and postoperative CBCT data were superimposed by point registration and automatic voxel-by-voxel registration at the unchanged craniomaxillofacial area by orthognathic surgery. On the superimposed images, LTW, APL, and CSA in the axial CV1, CV2, and CV3 were measured. The reference planes were measured using sagittal view cone-beam CT to set the retropalatal, retroglossal and glottis levels at upper airway. (A) The CV1, CV2, and CV3 planes, parallel to the Frankfort horizontal plane, are tangent to the most caudal medial projections of cervical vertebrae 1, 2, and 3, respectively. (B) On the superimposed image, T0, T1 APL and T0, T1 LTW were determined at CV1, CV2, and CV3 planes using axial view of cone-beam CT. (C) On the superimposed images, T0 and T1 CSA in the axial CV1, CV2, and CV3 were measured using Invivo 5. (D) The pre- and postoperative airway volume from retropalatal level (CV1) to glottis level (CV3) was measured in cubic millimeters using Airway Measurement tool of Invivo5. (APL = anterior-porterior length, CSA = cross-sectional area, LTW = lateral transverse width).

Reference planes, as based on midsagittal plane, are described in Figure 1A. The Frankfort plane was defined by the bilateral uppermost point on the bony external auditory meatus (porion) and lowest point on the right inferior borders of the bony orbit (orbitale). CV1, CV2, and CV3 planes were parallel to F-H plane and tangent to the most caudal medial projections of cervical vertebrae 1, 2, and 3, respectively. To evaluate changes of the pharyngeal airway, the largest transverse width (LTW), anteroposterior length (APL), and cross-sectional area (CSA) on each axial plane (CV1, CV2, CV3) were measured at T0 and T1 (Fig. 1B, C). Changes in the LTW, APL, and CSA were measured according to a method described using the Superimposition module of the Invivo 5 program.^[7]^ Pre- and postoperative CBCT data were superimposed by point registration and automatic voxel-by-voxel registration at the unchanged craniomaxillofacial area by orthognathic surgery. On the superimposed images, LTW, APL, and CSA in the axial CV1, CV2, and CV3 were measured. The pre- and postoperative airway volume from retropalatal level (CV1) to glottis level (CV3) was measured in cubic millimeters using the Airway Measurement tool of Invivo5 (Fig. 1D).

**Figure 2:**
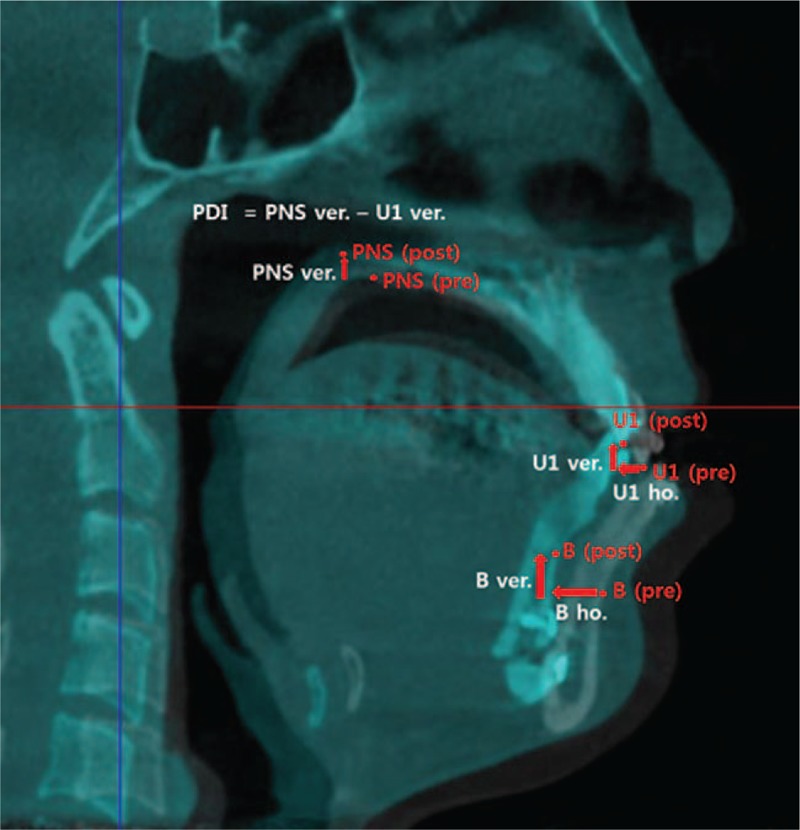
Landmarks measurements for surgical skeletal movements. The distances of maxillary and mandibular movements between before and after bimaxillary surgery were measured at PNS, U1, and B point (B points = innermost curvature from chin to alveolar bone junction, PDI = posterior differential impaction, PNS = posterior nasal spine, U1 = maxillary first molar root apices).

Using the same superimposition and coordinate system in which airway change was measured with, skeletal changes after bimaxillary surgery were evaluated. To assess movement of the maxilla and mandible, coordinate values of the U1 (incisal edge of right upper central incisor), B (B point), and PNS (posterior nasal spine) were calculated from the midsagittal view (Fig. 2) and positive values were assigned to posterior or superior movements. B point is defined as most concave point on mandibular symphysis, and posterior differential impaction represents the degree of maxillary clockwise rotation.

### 2.5 Statistical analysis

The results of airway volume, changes in anteroposterior diameter, transverse length, cross-sectional diameter, airway volume, and sleep parameters, such as AHI and ODI, are presented as mean values, and the significance of differences between preoperative and postoperative values of subjects who had bimaxillary surgeries was evaluated by analysis of a post hoc power analysis. The primary endpoint of this study was difference of sleep parameters between before and after surgery. A sample size determination was not possible because there was no study that provided sufficient data for power analysis. Thus, we performed a post hoc power analysis which indicated our study had over 80% power based on AHI and ODI values. Spearman's correlation analysis was performed to determine the correlation between airway configurations and skeletal movements. All analysis was performed with SPSS (version 18.0; SPSS Inc., Chicago, IL) for Windows software and *P* values less than .05 were considered statistically significant.

## 3 Results

### 3.1 Clinical characteristics of subjects with class III malocclusion

A total of 22 subjects diagnosed with class III malocclusion were included in the present study, including 5 men and 17 women. Le Fort I osteotomy and Sagital Split Ramus Osteotomy of the mandible were performed on all subjects as primary treatment for class III malocclusion. The mean age of the subjects was 22.1 years and mean body mass index was 22.5 kg/m^2^. Before bimaxillary surgery, endoscopic examination was carried out in all subjects and those with class III malocclusion were classified into Friedman stage according to the anatomic structure of the oropharynx. Twelve subjects were classified as Friedman stage I, 6 as Friedman stage II, 2 as Friedman stage III, and 2 as stage IV. No subjects were diagnosed with SBDs including OSA and primary snoring before surgery.

### 3.2 Cephalometric measurement of subjects with class III malocclusion

**Figure 3:**
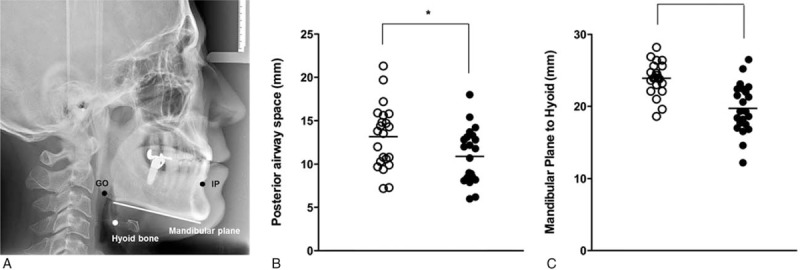
Changes of PAS and MPH in class III malocclusion subjects after bimaxillary surgery. Posterior airway space (PAS) and mandibular plane to hyoid (MPH) were measured using lateral cephalography. PAS was measured at the linear line from infradental point (IP) to gonion (GO) and MPH was defined as the distance from hypoid bone to mandibular plane (A). PAS and MPH measured using cephalography in class III malocclusion subjects, decreased significantly following bimaxillary surgery.

Pre- and postoperative values of PAS and MPH were measured using lateral cephalography and compared them to evaluate the degree of airway narrowing including retropalatal or retroglossal levels following surgery (Fig. 3A). The mean preoperative PAS value for the 22 subjects was 13.12 ± 3.7, whereas the mean postoperative PAS value was 10.91 ± 3.1, which was a statistically significant reduction (*P* < .05) (Fig. 3B). MPH value also decreased significantly following surgery, with a mean preoperative value of 23.91 ± 2.4 and a mean postoperative MPH value of 19.71 ± 3.4 (*P* < .05) (Fig. 3C). Overall, cephalometric measurements indicated that bimaxillary surgery caused upper airway narrowing in test subjects.

### 3.3 Volumetric analysis and measurement of the CSA of the upper airway

As a next step, changes in the diameter of the cross-sectional reference plane at CV1, CV2, and CV3 and upper airway volume were measured using 3D-CBCT images for more detailed analysis of upper airway narrowing. In general, mean total upper airway volume dropped from 10.2 to 8.0 cm^3^ after bimaxillary surgery. Changes in diameters and CSA are presented in Table 1. At the CV1 level, mean APL was 13.2 mm and LTW was 30.1 mm before surgery. After surgery, the mean LTW was significantly reduced by 26.1 mm. The mean CV1 CSA also significantly decreased from 349.1 to 278.7 mm^2^ following surgery, but the mean APL did not change much at the retropalatal level (Table 1). At the CV2 level, mean APL dropped significantly from 11.6 to 9.9 mm after surgery, and the mean CSA also decreased significantly from 251.7 to 195.7 mm^2^. The mean LTW was 25.5 mm before surgery and did not change much after surgery (Table 1). APL, LTW, and CSA at the CV3 were not smaller after surgery. The current findings showed that the attenuation of the APL and LTW appears to clinically significant after bimaxillary surgery in class III malocclusion patients, especially at the retropalatal and retroglossal level.

**Table 1:**
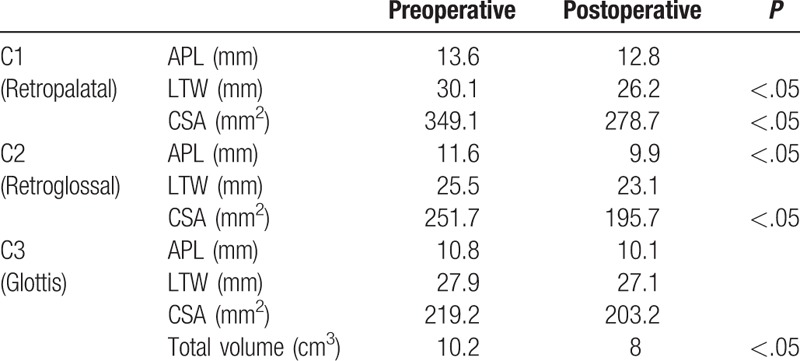
Pre- and postoperative alterations of anterior–posterior length (APL), lateral transverse width (LTW), cross-sectional area (CSA), and total upper airway volume of class III malocclusion subjects (N = 22) with bimaxillary surgery.

### 3.4 Sleep parameters after surgical correction of class III malocclusion

We performed sleep studies before and after bimaxillary surgery, and compared sleep parameters to assess whether reduced CSA and total upper airway volume were correlated with sleep apnea or snoring in class III malocclusion subjects. Preoperative sleep studies confirmed that all subjects had pAHI score under 5 and were not classified as having OSA. We also assessed the intensity of snoring sound in the subjects and the threshold for primary snoring was set at 40 dB. The mean dB of snoring sound before surgery was 26.7 ± 10.1 and no subjects complained subjectively of loud snoring during sleep.

**Figure 4:**
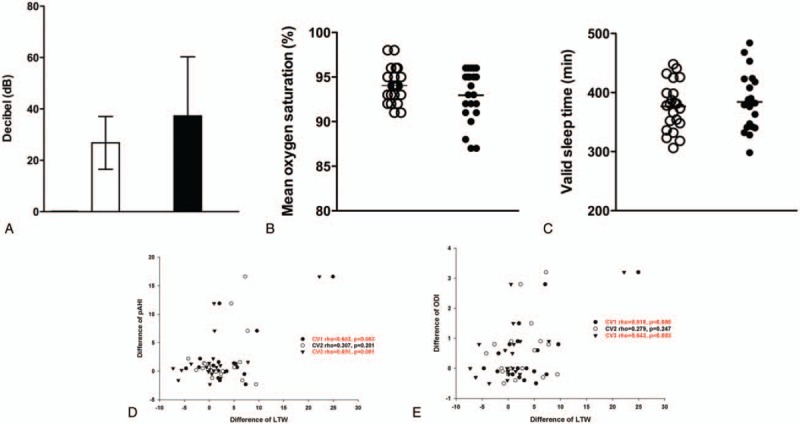
Changes in sleep parameters in class III malocclusion subjects after bimaxillary surgery. (A) Loudness of snoring, (B) mean oxygen saturation during sleep, and (C) valid sleep time were determined using watch-PAT in class III malocclusion subjects. Spearman's correlation analysis demonstrated that the amount of LTW (Largest transverse width) reduction at the retropalatal level (CV1) and epiglottis level (CV3) was significantly correlated with increase of AHI (D) and ODI (E).

Interestingly, pAHI scores increased to over 5 in 3 subjects and newly developed OSA was diagnosed after bimaxillary surgery in 13.6% of subjects with class III malocclusion (Table 2). One subject was diagnosed with mild OSA (pAHI = 10.5) and 2 with moderate OSA (pAHI 16.5 and 19.9). Loud snoring (over 40 dB), measured using the microphone of a watch-PAT during sleep, appeared in 6 subjects (27.2%) following surgery (Fig. 4A). Two subjects with OSA were also new snorers, so a total of 7 subjects had newly developed sleep-related symptoms such as snoring and apnea after bimaxillary surgery. Although both mean oxygen saturation during sleep and valid sleep time were not changed at 3 months after surgery (Fig. 4B, C), 3 subjects diagnosed with OSA following surgery showed below 90% mean oxygen saturation.

**Table 2:**
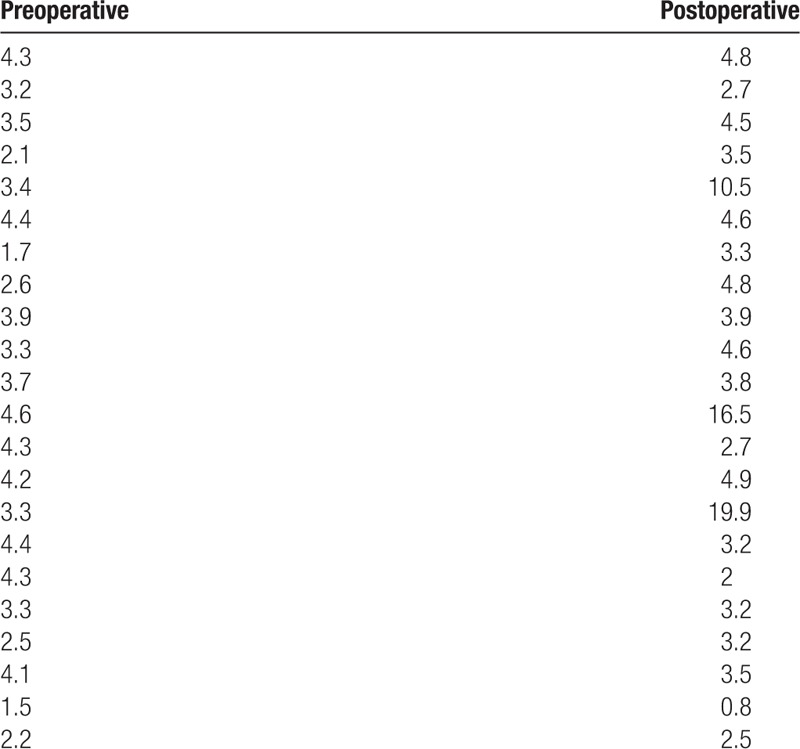
Pre- and postoperative apnea-hypopnea index (AHI) of class III malocclusion subjects (N = 22) with bimaxillary surgery.

Then, the changes of anterior–posterior and lateral diameters in the CV1, CV2, and CV3 were compared to sleep parameters through Spearman's correlation analysis. The analytic results showed that the amount of LTW reduction at CV1 and CV3 level was significantly correlated with increase of AHI and ODI (Fig. 4D, E). There was no correlation between snoring and pharyngeal airway changes. We assumed that alterations of PAS dimension and airway volume following bimaxillary surgery may influence sleep parameters of class III malocclusion subjects and induce objective snoring and sleep apnea following surgery.

### 3.5 Volumetric analysis and measurement of cross-sectional area of the upper airway in new snorers and OSA subjects

We next measured the APL and LTW of the cross-sectional reference plane at CV1, CV2, and CV3 and the upper airway volume in subjects with newly developed snoring and OSA following surgery (N = 7). Both LTW and CSA at the CV1 level were considerably smaller in new snorers and OSA subjects. The preoperative measurement of the mean LTW was 31.8 mm, which decreased to 26.2 mm after bimaxillary surgery (*P* < .05) (Table 3). In addition, CSA declined from 362.6 to 294.9 mm^2^ postoperatively (*P* < .05), with larger declines in new snorers and OSA subjects. In accordance with these findings, APL (12.1 mm preoperatively and 10.6 mm postoperatively) and CSA (266.1 mm^2^ preoperatively and 189.1 mm^2^ postoperatively) also declined more after bimaxillary surgery at the CV2 level (Table 3). Total upper airway volume was lowered further (11.7 cm^3^ preoperatively and 7.2 cm^3^ postoperatively) in new snorers and OSA subjects following surgery, but there were no significant changes in APL, LTW, or CSA at the CV3 level. Postoperative upper airway narrowing at the CV1 and CV2 levels was more striking in subjects with class III malocclusion who newly developed snoring and OSA after bimaxillary surgery.

**Table 3:**
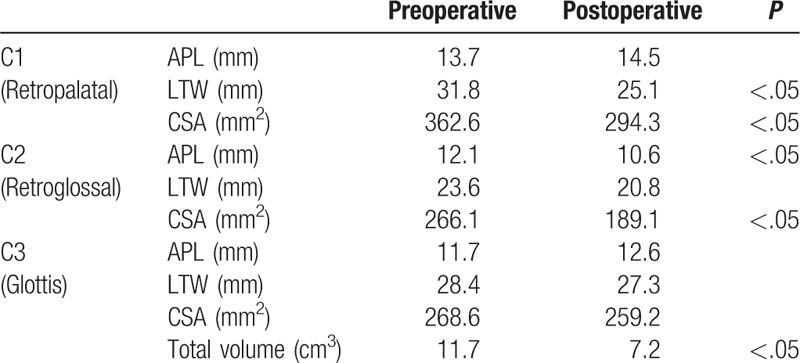
Pre- and postoperative alterations of anterior–posterior length (APL), lateral transverse width (LTW), cross-sectional area (CSA), and total upper airway volume of class III malocclusion subjects (N = 7) who had snoring and sleep apnea following bimaxillary surgery.

### 3.6 The skeletal movements and oropharyngeal examination of new snorers and OSA subjects following bimaxillary surgery

For the evaluation of skeletal movements of maxilla and mandible due to bimaxillary surgery in class III malocclusion, we calculated the horizontal and vertical distances of maxilla (U1) and mandible (B) at each landmark. The degree of maxillary clockwise rotation was also measured at PNS through posterior differential impaction (PDI) values.

**Figure 5:**
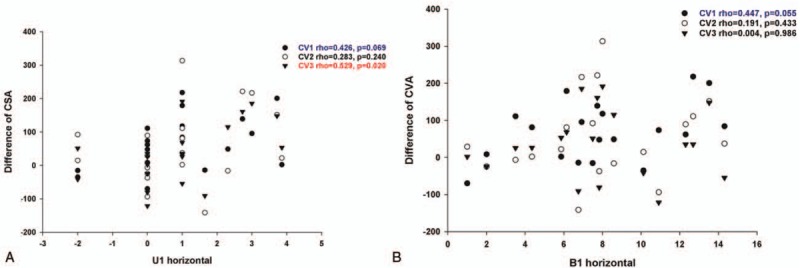
Measurement of airway diameter depending on posterior movement of maxilla and mandible after bimaxillary surgery. The amount of posterior movement of maxilla was correlated with a reduction of cross section area at the retropalatal level (CV1) and epiglottis level (CV3). (A) The amount of posterior movement of mandible was correlated with a reduction of cross section area at the retropalatal level (CV1). (B) There was no significant correlation between CV2 and posterior movements of maxilla or mandible.

The results showed that the amount of posterior movement of maxilla was correlated with a reduction of CSA at CV1 and CV3 (Fig. 5A). The amount of posterior movement of mandible was correlated with a reduction of CSA at CV1 (Fig. 5B). Interestingly, mean skeletal movements of horizontal and vertical distances were 0.91 and 1.61 mm, and PDI value was 2.67 mm in class III malocclusion subjects without development of snoring and apnea. However, mean distance of maxillary and mandibular movements was relatively higher (horizontal distance: 1.23 mm, vertical distance: 2.41 mm), and PDI value (3.38 mm) was considerably greater in new snorers and OSA subjects (Table 4). In addition, new snorers and OSA class III malocclusion subjects following bimaxillary surgery showed larger vertical movements of mandible (7.48 vs 5.48 mm in subjects without snoring and apnea).

**Table 4:**
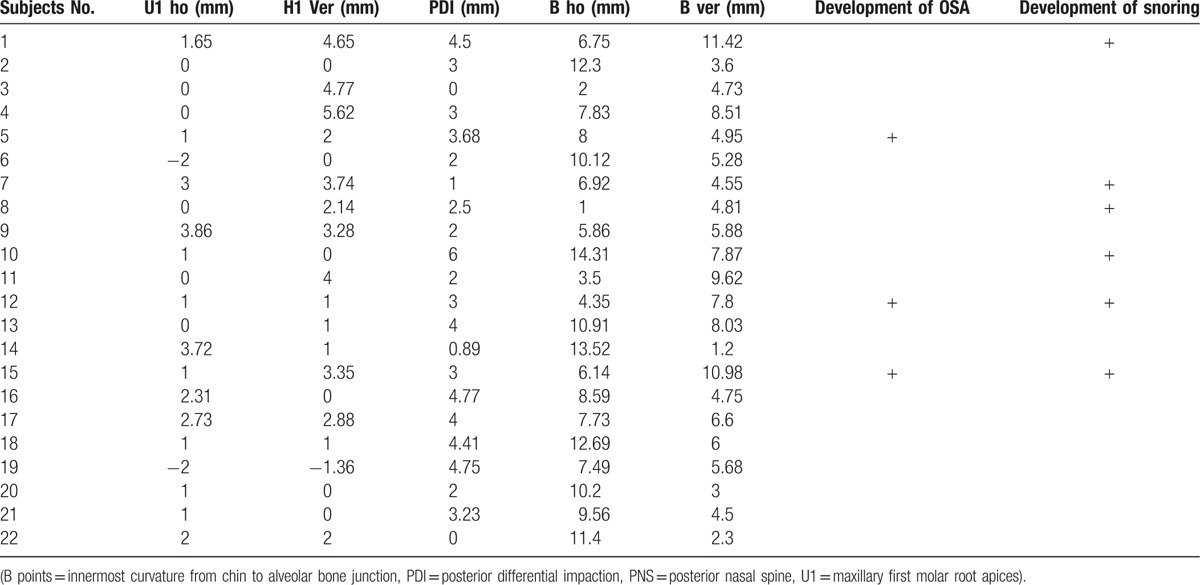
The summarized data for skeletal movements of subjects after bimaxillary surgery.

Finally, we investigated the anatomic structures of the oropharynx in class III malocclusion to assess if oropharyngeal examination significantly differed in the patients who had newly developed snoring and OSA. We classified the subjects by preoperative Friedman stage according to their palatal and tonsillar grade. Of the 7 subjects, 2 were stage III and 2 were stage IV. Soft palate redundancy appeared to be significant in these 4 subjects with class III malocclusion. We found that 2 classified as Friedman stage III and IV began snoring following bimaxillary surgery and 3 of them were diagnosed with OSA after surgical correction for their malocclusion. These data suggest that the prevalence of snoring or OSA in class III malocclusion is dependent on the degree of skeletal movements of maxilla or mandible and that snoring and OSA are more likely to occur after surgery if the subject has a redundant soft palate.

## 4 Discussion

Through this prospective study, we found that bimaxillary surgery caused upper airway narrowing at the retropalatal or retroglossal level and reduced total upper airway volume triggering snoring and OSA in class III malocclusion subjects. The current clinical findings also propose that simultaneous setback of the maxilla and mandible increases the probability of causing such a SBD and airway configuration and sleep study need to be performed before bimaxillary surgery in class III malocclusion subjects

Dentofacial measurement results about class III malocclusion usually present with skeletal discrepancies, such as a prognathic mandible with or without a protruding maxilla.^[2,3,8]^ The surgical treatment of class III malocclusions, including mandibular setback, affects pharyngeal anatomic structures and can cause volumetric or dimensional changes in upper airway narrowing.^[7,8]^ Recently, narrowing of the upper airway after orthognathic surgeries has received attention in orthognathic researches, with a focus on patients who receive bimaxillary surgery and may develop SBDs including OSA due to structural alterations of bone, muscle, and soft tissue around the pharynx.^[6,9,20,21]^

We assessed AHI and intensity of snoring pre- and postoperatively through prospective investigation in class III malocclusion patients scheduled to have bimaxillary surgery, and found that the prevalence of newly developed snoring was 27.2% and OSA was 13.6% following surgery. Our findings indicate that dentofacial repositioning by bimaxillary surgery can lead to objective snoring or OSA in subjects with class III malocclusion and sleep studies would be necessary to describe more definite changes of sleep parameters before or following bimaxillary surgery in class III malocclusion.

Upper airway narrowing may contribute to the pathologic conditions of OSA and some evidence suggests significant improvement in symptoms or sleep parameters based on correction of upper airway collapse in OSA patients.^[22–24]^ Therefore, detailed imaging analysis about upper airway narrowing would be required to predict the prevalence of OSA and to establish its therapeutic plans. Good correlation between airway dimensions measured on CBCT has been reported, and the usefulness of cephalometric analysis such as, PAS and MPH, has been validated for measurement of upper airway size.^[25,26]^ We described alterations of upper airway volume and dimensional extent at the retropalatal, retroglossal, and glottis levels following bimaxillary surgery using lateral cephalometric radiographs and CBCT. Airway narrowing or increased collapsibility at both the oropharynx and tongue base is most common in OSA patients. The decrease of airway patency can promote more negative intraluminal pressure within the upper airway and provoke an increase in airway collapse at the level of oropharynx or hypopharynx.^[27]^ In addition, airway narrowing may cause or aggravate symptoms of OSA due to severe airflow resistance.^[28,29]^ We assumed that bimaxillary surgery in class III malocclusion subjects drags the oropharynx and hypopharynx in the posterior direction, and that the posterior-displaced oropharynx, including the tongue base, narrows the retrogrossal dimension and decreases the PAS in the similar vector. Surgery resulted in a significant reduction in APL and CSA at the retroglossal level. Retropalatal dimension was also reduced significantly following bimaxillary surgery and CSA was decreased at the level of the palate area. These narrowed upper airway and anatomic alterations at the retropalatal and retroglossal level caused a significant reduction of total upper airway volume, resulting in snoring and sleep apnea in some subjects. Our findings are in agreement with previous studies that also reported considerable constriction of the PAS at the oropharyngeal level, including the tongue base, after mandibular setback.^[8–10,24]^ The narrowing upper airway after surgery reflects the change of dentofacial morphology closely, and a decrease in the upper airway leads to changes in sleep parameters of class III malocclusion subjects.

Interestingly, the reduction in dimension at the retropalatal and retroglossal levels and total upper airway volume were more extensive in subjects who developed snoring and OSA following surgery. In particular, more increase of horizontal and vertical movements of maxilla or mandible were observed in class III malocclusion subjects developing snoring and apnea after orthognathic surgery, and a redundant uvula or soft palate was more often present in subjects with postoperative snoring and apnea. A strong correlation between the amount of mandibular setback and the decrease in the PAS was reported, and the low magnitude of mandibular posterior repositioning may explain the absence of significant changes in the PAS.^[30–34]^ Our data showed that the posterior movement of the maxilla and the mandible narrowed the pharyngeal airway and reduction of pharyngeal airway might be related with the development of snoring or sleep apnea. The 7 subjects with postoperative snoring and OSA in our study may also have been influenced by a larger magnitude of maxillar or mandibular backward positioning. Therefore, cephalometric analysis should be carefully evaluated before surgical correction of class III malocclusion to minimize the risk of postoperative SBDs and there is a high risk of excessive setback of the maxilla and mandible to enhance the probability of SBDs. Although the present study has a limitation that the sample size of the study was small and follow-up period was to short, despite designing of prospective study, our data suggest that sleep study might be critical for class III malocclusion subjects who underwent bimaxillary surgery, and accurate evaluation of upper airway in oropharynx before starting bimaxillary surgery may also provide the clinical information to predict the possibility of SBD after operation.

In conclusion, postoperative narrowing of the upper airway and reduction of total upper airway volume were induced by bimaxillary surgery and caused snoring and OSA following surgery in some class III malocclusion subjects. Delicate airway configuration and surgical planning should be based on the upper airway to prevent SBDs in class III malocclusion subjects and a plan should be established so that the maxilla can be moved forward when performing surgery through anterior segmental osteotomy or orthodontic premolar extraction in cooperation with the orthodontist.

## References

[1] Ghassemi M Jamilian A Becker JR Soft-tissue changes associated with different surgical procedures for treating class III patients. J Orofac Orthop 2014;75:299–307. 24996857 10.1007/s00056-014-0224-x

[2] Burden DJ Hunt O Johnston CD Psychological status of patients referred for orthognathic correction of skeletal II and III discrepancies. Angle Orthod 2010;80:43–8. 19852638 10.2319/022709-114.1 PMC8978754

[3] Johnston C Burden D Kennedy D Class III surgical-orthodontic treatment: a cephalometric study. Am J Orthod Dentofacial Orthop 2006;130:300–9. 16979487 10.1016/j.ajodo.2005.01.023

[4] Fernández-Ferrer L Montiel-Company JM Pinho T Effects of mandibular setback surgery on upper airway dimensions and their influence on obstructive sleep apnoea—a systematic review. J Craniomaxillofac Surg 2015;43:248–53. 25547214 10.1016/j.jcms.2014.11.017

[5] Al-Nawas B Kämmerer PW Hoffmann C Influence of osteotomy procedure and surgical experience on early complications after orthognathic surgery in the mandible. J Craniomaxillofac Surg 2014;42:e284–8. 24289870 10.1016/j.jcms.2013.10.007

[6] Ranna V Kellesarian SV Feng C Influence of the orthognathic surgical procedure Le Fort I osteotomy on the vascularity and neurosensory response of the dental pulp: a systematic review. Quintessence Int 2016;47:677–86. 27341469 10.3290/j.qi.a36386

[7] Gokce SM Gorgulu S Gokce HS Evaluation of pharyngeal airway space changes after bimaxillary orthognathic surgery with a 3-dimensional simulation and modeling program. Am J Orthod Dentofacial Orthop 2014;146:477–92. 25263151 10.1016/j.ajodo.2014.06.017

[8] Li YM Liu JL Zhao JL Morphological changes in the pharyngeal airway of female skeletal class III patients following bimaxillary surgery: a cone beam computed tomography evaluation. Int J Oral Maxillofac Surg 2014;43:862–7. 24697918 10.1016/j.ijom.2014.03.009

[9] Gokce SM Gorgulu S Gokce HS Changes in posterior airway space, pulmonary function and sleep quality, following bimaxillary orthognathic surgery. Int J Oral Maxillofac Surg 2012;41:820–9. 22476009 10.1016/j.ijom.2012.01.003

[10] Foltán R Hoffmannová J Donev F The impact of Le Fort I advancement and bilateral sagittal split osteotomy setback on ventilation during sleep. Int J Oral Maxillofac Surg 2009;38:1036–40. 19560899 10.1016/j.ijom.2009.06.001

[11] Lee JY Kim YI Hwang DS Effect of maxillary setback movement on upper airway in patients with class III skeletal deformities: cone beam computed tomographic evaluation. J Craniofac Surg 2013;24:387–91. 23524700 10.1097/SCS.0b013e31827fef0f

[12] Bilston LE Gandevia SC Biomechanical properties of the human upper airway and their effect on its behavior during breathing and in obstructive sleep apnea. J Appl Physiol 2014;116:314–24. 23823151 10.1152/japplphysiol.00539.2013

[13] Chung S Yoon IY Shin YK Endotherlial dysfunction and inflammatory reactions of elderly and middle-aged men with obstructive sleep apnea syndrome. Sleep Breath 2009;13:11–7. 18777130 10.1007/s11325-008-0210-x

[14] Gozal D Kheirandish-Gonzal L Cardiovascular morbidity in obstructive sleep apnea: oxidative stress, inflammation, and much more. Am J Respir Crit Care Med 2008;177:369–75. 17975198 10.1164/rccm.200608-1190PP PMC2258438

[15] Marshall NS Wong KK Liu PY Sleep apnea as an independent risk factor for all-cause mortality: the Busselton Health Study. Sleep 2008;31:1079–85. 18714779 PMC2542953

[16] Parish JM Somer VK Obstructive sleep apnea and cardiovascular disease. Mayo Clin Proc 2004;79:1036–46. 15301332 10.4065/79.8.1036

[17] Peppard PE Young T Palta M Prospective study of the association between sleep-disordered breathing and hypertension. N Engl J Med 2000;342:1378–84. 10805822 10.1056/NEJM200005113421901

[18] Pereira-Filho VA Castro-Silva LM de Moraes M Cephalometric evaluation of pharyngeal airway space changes in class III patients undergoing orthognathic surgery. J Oral Maxillofac Surg 2011;69:e409–15. 21757274 10.1016/j.joms.2011.02.132

[19] Jakobsone G Neimane L Krumina G Two- and three-dimensional evaluation of the upper airway after bimaxillary correction of Class III malocclusion. Oral Surg Oral Med Oral Pathol Oral Radiol Endod 2010;110:234–42. 20580280 10.1016/j.tripleo.2010.03.026

[20] Park CY Hong JH Lee JH Clinical effect of surgical correction for nasal pathology on the treatment of obstructive sleep apnea syndrome. PLoS One 2014;9:e98765. 24896824 10.1371/journal.pone.0098765 PMC4045850

[21] Sher AE Schechtman KB Piccirillo JF The efficacy of surgical modifications of the upper airway in adults with obstructive sleep apnea syndrome. Sleep 1996;19:156–77. 8855039 10.1093/sleep/19.2.156

[22] Peppard PE Young T Barnet JH Increased prevalence of sleep-disordered breathing in adults. Am J Epidemiol 2013;177:1006–14. 23589584 10.1093/aje/kws342 PMC3639722

[23] Peppard PE Young T Palta M Prospective study of the association between sleep-disordered breathing and hypertension. N Engl J Med 2000;342:1378–84. 10805822 10.1056/NEJM200005113421901

[24] Demetriades N Chang DJ Laskarides C Effects of mandibular retropositioning, with or without maxillary advancement, on the oro-naso-pharyngeal airway and development of sleep-related breathing disorders. J Oral Maxillofac Surg 2010;68:2431–6. 20663598 10.1016/j.joms.2010.02.033

[25] Pittayapat P Limchaichana-Bolstad N Willems G Three-dimensional cephalometric analysis in orthodontics: a systematic review. Orthod Craniofac Res 2014;17:69–91. 24373559 10.1111/ocr.12034

[26] Katkar RA Kummet C Dawson D Comparison of observer reliability of three-dimensional cephalometric landmark identification on subject images from Galileos and i-CAT cone beam CT. Dentomaxillofac Radiol 2013;42: 20130059. 10.1259/dmfr.20130059 PMC3828023 23833319

[27] Schwartz RN Payne RJ Forest VI The relationship between upper airway collapse and the severity of obstructive sleep apnea syndrome: a chart review. J Otolaryngol Head Neck Surg 2015;44:32–8. 26334998 10.1186/s40463-015-0086-2 PMC4558755

[28] Rodrigues MM Real Gabrielli MF Watanabe ER Correlation between the Friedman Staging System and the upper airway volume in patients with obstructive sleep apnea. J Oral Maxillofac Surg 2015;73:162–7. 25443383 10.1016/j.joms.2014.07.030

[29] Jordan AS Cori JM Dawson A Arousal from sleep does not lead to reduced dilator muscle activity or elevated upper airway resistance on return to sleep in healthy individuals. Sleep 2015;38:53–9. 25325511 10.5665/sleep.4324 PMC4262956

[30] Santagata M Tozzi U Lamart E Effect of orthognathic surgery on the posterior airway space in patients affected by skeletal class III malocclusion. J Maxillofac Oral Surg 2015;14:682–6. 26225062 10.1007/s12663-014-0687-8 PMC4510090

[31] Kawakami M Yamamoto K Fujimoto M Changes in tongue and hyoid positions, and posterior airway space following mandibular setback surgery. J Craniomaxillofac Surg 2005;33:107–10. 15804589 10.1016/j.jcms.2004.10.005

[32] Kawakami M Yamamoto K Noshi T Effect of surgical reduction of the tongue on dentofacial structure following mandibular setback. J Oral Maxillofac Surg 2004;62:1188–92. 15452803 10.1016/j.joms.2004.06.032

[33] Canellas JV Barros HL Medeiros PJ Effects of surgical correction of class III malocclusion on the pharyngeal airway and its influence on sleep apnoea. Int J Oral Maxillofac Surg 2016;45:1508–12. 27688168 10.1016/j.ijom.2016.09.002

[34] Scherer JM Sheats RD Phillips C Class III bimaxillary orthognathic surgery and sleep disordered breathing outcomes. J Dental Sleep Med 2015;2:157–62.

